# Development and validation of an open-source tool for opportunistic screening of osteoporosis from hip CT images: a multicentre study of 978 hips

**DOI:** 10.1302/2046-3758.129.BJR-2023-0115.R1

**Published:** 2023-09-20

**Authors:** Keisuke Uemura, Yoshito Otake, Kazuma Takashima, Hidetoshi Hamada, Takashi Imagama, Masaki Takao, Takashi Sakai, Yoshinobu Sato, Seiji Okada, Nobuhiko Sugano

**Affiliations:** 1 Department of Orthopaedic Medical Engineering, Graduate School of Medicine, Osaka University, Suita, Japan; 2 Division of Information Science, Graduate School of Science and Technology, Nara Institute of Science and Technology, Ikoma, Japan; 3 Department of Orthopaedics, Graduate School of Medicine, Osaka University, Suita, Japan; 4 Department of Orthopaedics, Graduate School of Medicine, Yamaguchi University, Ube, Japan; 5 Department of Bone and Joint Surgery, Graduate School of Medicine, Ehime University, Toon, Japan

**Keywords:** Osteoporosis, Artificial intelligence, Bone mineral density, hips, bone mineral density (BMD), proximal femur, dual-energy X-ray absorptiometry, femora, hip surgery, clinicians, lesser trochanter, correlation coefficients

## Abstract

**Aims:**

This study aimed to develop and validate a fully automated system that quantifies proximal femoral bone mineral density (BMD) from CT images.

**Methods:**

The study analyzed 978 pairs of hip CT and dual-energy X-ray absorptiometry (DXA) measurements of the proximal femur (DXA-BMD) collected from three institutions. From the CT images, the femur and a calibration phantom were automatically segmented using previously trained deep-learning models. The Hounsfield units of each voxel were converted into density (mg/cm^3^). Then, a deep-learning model trained by manual landmark selection of 315 cases was developed to select the landmarks at the proximal femur to rotate the CT volume to the neutral position. Finally, the CT volume of the femur was projected onto the coronal plane, and the areal BMD of the proximal femur (CT-aBMD) was quantified. CT-aBMD correlated to DXA-BMD, and a receiver operating characteristic (ROC) analysis quantified the accuracy in diagnosing osteoporosis.

**Results:**

CT-aBMD was successfully measured in 976/978 hips (99.8%). A significant correlation was found between CT-aBMD and DXA-BMD (r = 0.941; p < 0.001). In the ROC analysis, the area under the curve to diagnose osteoporosis was 0.976. The diagnostic sensitivity and specificity were 88.9% and 96%, respectively, with the cutoff set at 0.625 g/cm^2^.

**Conclusion:**

Accurate DXA-BMD measurements and diagnosis of osteoporosis were performed from CT images using the system developed herein. As the models are open-source, clinicians can use the proposed system to screen osteoporosis and determine the surgical strategy for hip surgery.

Cite this article: *Bone Joint Res* 2023;12(9):590–597.

## Article focus

- To develop a fully automated system to measure the bone mineral density (BMD) of the proximal femur from CT images.
- To validate the accuracy of the developed system in a multicentre study.

## Key messages

- By using three deep learning-based models, the BMD of the proximal femur was accurately measured from CT images.
- The developed system can be used to opportunistically screen for osteoporosis.
- The developed system can aid surgeons in selecting the type of implant for hip surgery.

## Strengths and limitations

- A fully automated system to measure the BMD of the proximal femur from CT images was developed and validated in a large cohort.
- Open-source models were used for the analysis.
- More experiments may be needed to further confirm the robustness of the developed system.

## Introduction

Screening and diagnosis of osteoporosis and subsequent adequate treatment are crucial for preventing fragile bone fractures that reduce quality of life. As recommended by the World Health Organization and several guidelines, osteoporosis is typically diagnosed by measuring the bone mineral density (BMD) of the proximal femur or lumbar region by dual-energy X-ray absorptiometry (DXA).^1-3^ However, as osteoporosis is generally asymptomatic, patients are less likely to undergo DXA, leading to underdiagnosis and undertreatment of osteoporosis.^4^

To improve the diagnostic rate of osteoporosis and enable treatment before fragile bone fractures develop, several studies have examined the use of other imaging methods for screening osteoporosis.^5^ For example, screening via pulse-echo ultrasound of the lower leg,^6,7^ ultrasonography of the calcaneus (i.e. quantitative ultrasonography),^8^ and radiography of the metacarpal bone (i.e. microdensitometry) and wrist have been reported.^8,9^ Yet, the relatively low accuracy of these methods in diagnosing osteoporosis and limited availability of the equipment and methods necessary for clinical investigation remain an issue. Other screening methods include quantitative CT (QCT) images for BMD analysis. A commercially available software (e.g. QCT Pro; Mindways Software, USA) has been developed to estimate BMD measured by DXA (DXA-BMD) from CT images,^10^ and based on the very high correlation coefficient (r > 0.9) between areal BMD measured from QCT images and DXA-BMD,^11-15^ CT-measured BMD is listed on the official positions by the International Society for Clinical Densitometry (ISCD).^15,16^ However, as commercial software adds to the cost, adoption of CT for measuring BMD and screening osteoporosis remains limited in clinical practice. Recently, an open-source software to measure the BMD from hip CT images was reported.^17^ However, the software was only verified in a small sample (75 cases) from one institution, and manual annotation of the landmarks at the proximal femur was necessary. Thus, developing an open-source fully automated system that can measure BMD from clinically acquired hip CT images was deemed necessary to help surgeons to select implants for hip arthroplasty (e.g. cemented or cementless stem) and suggest treatment for patients with osteoporosis. This study aimed to develop a fully automated system for quantifying proximal femur BMD from hip CT images and validate the accuracy of the developed system in a multicentre study to confirm its versatility.

## Methods

### Participants

Ethical approval was obtained from the institutional review boards of all institutions participating in this retrospective study. The study participants consisted of a total of 857 patients (978 hips) who underwent hip surgery at three institutions (A: Osaka University; B: Yamaguchi University; and C: Ando Hospital) (Table I). The sample was composed of 189 male and 668 female patients, and the majority of the patients underwent surgery for osteoarthritis in institutions A and B, whereas it was for proximal femoral fracture in institution C. Patients who had metal implants in the spine or femur were excluded. Of the 978 hips, 377 (38.5%) were diagnosed as ‘osteoporotic’, according to the T-score for women calculated from the BMD measurement of the total proximal femoral region using DXA. These T-scores were standardized by a correction method proposed by the Japan Osteoporosis Society,^1^ which refers to the guidelines of the ISCD.^18^

**Table I.** Demographics of the patients from three institutions.

Demographic	A	B	C	Overall
Patients/hips, n (%)	315/315	167/288	375/375	857/978
Sex (male/female), n (%)	59/256	36/131	94/281	189/668
Mean age, yrs (SD)	57.3 (15.3)	65.5 (11.7)	82.6 (9.6)	70.0 (16.8)
Mean height, cm (SD)	157.9 (12.1)	154.9 (8.5)	153.5 (9.4)	155.4 (9.1)
Mean weight, kg (SD)	58.7 (12.7)	59.2 (12.7)	49.4 (10.4)	54.7 (12.5)
Mean BMI, kg/m^2^ (SD)	24.6 (4.3)	24.6 (4.3)	20.9 (3.7)	22.5 (4.2)
**Hip disease, n of patients**				
OA	263	148	2	413
ONFH	47	19		66
RDC	4			4
ALT	1			1
PFF			373	373
Osteoporosis, n of hips (%)	51 (16.2)	46 (16.0)	280 (74.7)	377 (38.5)

ALT, acetabular labrum tear; OA, osteoarthritis; ONFH, osteonecrosis of the femoral head; PFF, proximal femoral fracture; RDC, rapidly destructive coxopathy; SD, standard deviation.

### Image acquisition

Preoperative hip CT images acquired for surgical planning were analyzed in this study. During CT image acquisition, a calibration phantom made of urethane foam containing four hydroxyapatite rods (B-MAS200; Kyoto Kagaku, Japan) was placed under the patient’s hip. For the three institutions, five CT models from three manufacturers were used for image acquisition (Table II). A standard clinical protocol with a matrix of 512 × 512, a tube voltage of 120 kVP, and voxel sizes ranging from (0.545 mm to 0.977 mm) × (0.545 mm to 0.977 mm) × (0.625 mm to 2.0 mm) was used to acquire all CT images. The pixel size of the CT images was set to include the pelvis and femur within the region of interest (ROI), and the slice interval at institution C was discussed between each surgeon (KU, KT, HH) and the radiology department for their preference in the 3D reconstruction of the bone on a software with multiplanar planning (ZedHip; Lexi Co, Japan). A convolutional kernel representing bone or soft-tissue kernel was used to reconstruct CT images.

**Table II.** CT images and dual-energy X-ray absorptiometry acquired at three institutions.

Factor	A	B			C	
CT manufacturer (model)	GE (Optima CT660)	GE (Optima CT660)	Toshiba (Aquilion Precision)	Siemens (SOMATOM Force)	Toshiba (Activion 16)	GE (Revolution Maxima)
CT convolution kernel	STD	STD	FC03	B40/B44	FC03/FC30	STD
CT voxel size, mm	(0.545 to 0.820) × (0.545 to 0.820) × (1.25)	(0.703 to 0.742) × (0.703 to 0.742) × (1.25)	(0.625 to 0.977) × (0.625 to 0.977) × (1.0)	(0.742 to 0.977) × (0.742 to 0.977) × (1.0)	(0.624 to 0.972) × (0.624 to 0.972) × (1.0 or 2.0)	(0.625 to 0.977) × (0.625 to 0.977) × (0.625 or 1.25 or 2.0)
N of hips	315	6	196	3/83	129/103	143
DXA manufacturer (model)	Hologic (Discovery A)	Hologic (Horizon A)			GE (Prodigy Fuga)	
Median CT–DXA duration, days (IQR)	7 (1 to 14)	0 (0 to 16)			10 (7 to 14)	

DXA, dual-energy X-ray absorptiometry; GE, General Electric Healthcare Japan; IQR, interquartile range; Toshiba, Toshiba Medical Systems.

For each patient before or after the CT acquisition, DXA images of the proximal femur were acquired using three DXA models from two manufacturers (Table II). Specifically, for patients who underwent hip arthroplasty, DXA images were acquired preoperatively to determine the stem implant type (i.e. cement or cementless stem). Conversely, for patients with proximal femoral fracture, DXA was acquired postoperatively to determine the osteoporosis treatment method. As BMD values differ between the manufacturers, a previously reported equation was used to convert the value measured in GE’s DXA (GE Healthcare Japan, Japan) to that measured in Hologic’s DXA (Hologic Japan, Japan).^19^ The median interval between the acquisitions of CT and DXA images was eight days (interquartile range (IQR) 1 to 14).

### Measurement methods

**Fig. 1:**
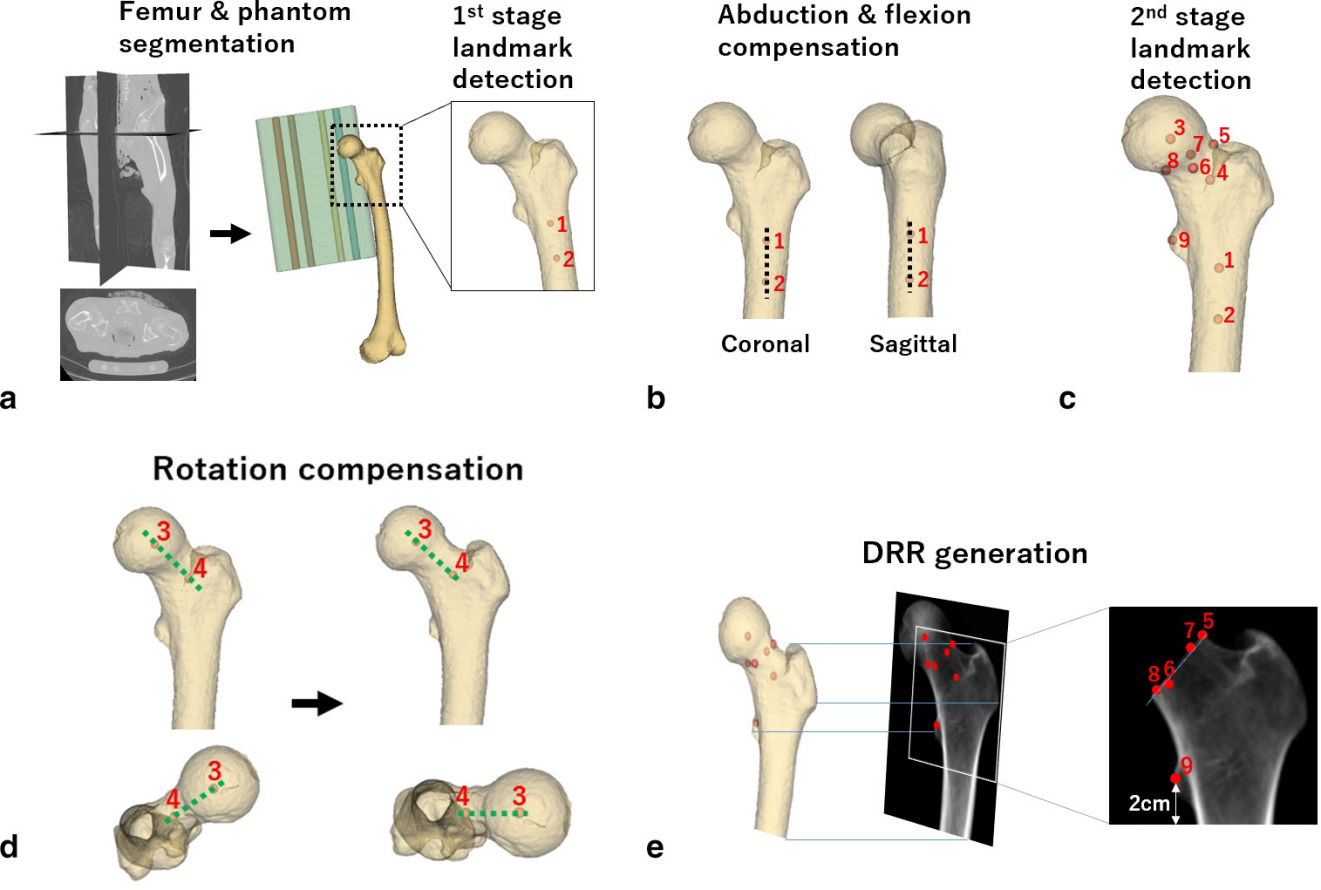
Flowchart of the proposed method for measuring the CT-aBMD for a left femur. a) The femur and phantom were segmented from the CT images, and two landmarks ((1) 2 cm distal from the lesser trochanter; and (2) 5 cm distal from the lesser trochanter) were selected. b) The femoral volume was rotated to the neutral position on the coronal and sagittal planes. c) Additional seven landmarks were selected, namely: (3) head centre; (4) neck centre; (5 to 8) head-neck junction of the superior, anterior, posterior, and inferior regions; and (9) tip of the lesser trochanter. d) The femoral volume was rotated on the axial plane to the neutral position. e) The femoral volume in the neutral position was projected onto the coronal plane and was cropped using the projected landmarks to measure CT-aBMD. The black dotted line in b) indicates the shaft axis, and the green dotted line in d) indicates the neck axis. aBMD, areal bone mineral density; DRR, digitally reconstructed radiograph.

On the basis of a previous report, deep learning was used to develop a system to measure the BMD of the proximal femur.^17^ Specifically, models previously reported to segment the calibration phantom and the femur were used,^17,20^ and a new model to detect the landmarks of the proximal femur was developed and used (Figure 1).

### Femur and phantom segmentation from CT images

A publicly available Bayesian U-Net model, which is a convolutional neural network for semantic segmentation that was previously reported and validated (Figure 1a), was used to perform femur and phantom segmentation from the CT images for each case.^21^ Briefly, the femoral segmentation model was trained on the manual segmentation of bilateral femora in 120 cases, and the phantom segmentation model was trained on the manual segmentation of the phantom in 40 cases. The accuracy of femoral segmentation was reported as a mean Dice coefficient^22^ of 0.985 (standard deviation (SD) 0.0065) and a mean symmetric surface distance (MSD)^23^ of 0.175 mm (SD 0.084),^21^ whereas the accuracy of the phantom segmentation was reported as a median Dice coefficient of 0.977 (IQR 0.963 to 0.986) and a MSD of 0.116 mm (IQR 0.084-0.193).^20^ Use of phantom segmentation enabled determination of a linear correlation equation between the mean Hounsfield units (HUs) and the known densities of the phantom (0, 50, 100, 150, and 200 mg/cm^3^ in hydroxyapatite). Subsequently, using the correlation equation, the HUs of each voxel were converted into density (mg/cm^3^).

### Development of a landmark detection model

To define the proximal femoral region for measuring BMD and account for the variations in patient positioning during CT, a deep-learning model that automatically selects the landmarks of the proximal femur from the CT images was developed. Specifically, to construct the training data, an orthopaedic surgeon with 15 years of experience (KU) manually selected nine landmarks: (1 to 2) 2 and 5 cm distal from the tip of the lesser trochanter; (3) head centre; (4) neck centre; (5 to 8) head-neck junction of the superior, anterior, posterior, and inferior regions; and (9) tip of the lesser trochanter, from CT images of institution A (315 hips) using software that allowed for multiplanar reconstruction (3D template; Kyocera, Japan). As pre-processing, CT images were cropped to include the proximal femoral region using the segmentation information of the femur and were downsampled to 128 × 128 × 128. During the training phase, data augmentation was conducted so that the model could account for the variations in patient positioning. Specifically, translation by (-25%, +25%) of the matrix size, rotation of (-30°, +30°) around the x-axis, rotation of (-15°, +15°) around the y- and z-axes, and scaling of (-20%, +20%) were conducted.

### CT-aBMD measurement

The landmarks were selected in two stages to account for substantial variability in patient positioning during CT. For example, patients from institution C had a large positioning variation because of pain caused by the fracture on the contralateral side. First, landmarks of 2 and 5 cm distal from the tip of the lesser trochanter were selected by the landmark detection model to account for femoral flexion and abduction during image acquisition (Figure 1a). Subsequently, the CT volume was rotated to the neutral position (Figure 1b), and landmark detection for the nine landmarks was performed again (Figure 1c). By using the landmarks of head and neck centres, the CT volume was rotated on the axial plane to account for rotation and compensate for femoral anteversion (Figure 1d). The rotated volume was projected onto the coronal plane using trilinear interpolation with a step length of 1 mm,^24^ and a digitally reconstructed radiograph (DRR) similar to the DXA images was generated (Figure 1e). The landmarks were also projected onto the coronal plane and used to isolate the proximal femoral region in the DRR (Figure 1e). The distal end of the proximal femur was defined as 2 cm distal from the tip of the lesser trochanter. The mean density of the proximal femoral region was measured and converted to units in DXA (g/cm^2^) to enable comparison with DXA-BMD.^17^

### Comparison of BMD measurements in CT and DXA

CT-aBMD correlated to DXA-BMD of the total proximal femur, and the correlation coefficients were calculated. Further, the ability of CT-aBMD to estimate the DXA-BMD was evaluated using the Bland–Altman plots, and absolute differences were also calculated. To diagnose osteoporosis (classified by the T-score in DXA-BMD), the receiver operating characteristic (ROC) curve was analyzed for CT-aBMD. As a subanalysis, the absolute differences between CT-aBMD and DXA-BMD were compared across institutions and CT manufacturers.

### Accuracy of the landmark detection model and comparison of CT-aBMD measured from manually selected landmarks

The accuracy of the landmark detection model was validated internally and externally. For internal validation, fourfold cross-validation was performed on the training dataset (315 hips). For external validation, nine landmarks were manually selected for 103 hips acquired using Activion 16 (Toshiba Medical Systems, Japan) in institution C and reconstructed with a convolution kernel of FC30 (Table II).

By using the landmarks selected manually and automatically, the 3D distance between the landmarks was calculated. Further, the angles (abduction, flexion, and rotation) to rotate the images to the neutral position were calculated and compared. Finally, the CT-aBMD measured using the manually and automatically selected landmarks was compared.

### Statistical analysis

The Shapiro–Wilk test was performed to confirm data normality. Normally distributed data were expressed as means (SDs), while non-normally distributed data were expressed as medians and IQRs. The correlation between two variables was assessed with a Pearson correlation coefficient, and r > 0.8 was considered to be very strong.^25^ To diagnose osteoporosis, the area under the curve (AUC), optimal cutoff value, sensitivity, and specificity for the ROC curve analysis were calculated. The Kruskal–Wallis test was performed to compare absolute differences across institutions and CT manufacturers. All statistical analysis was performed using MATLAB v9.10 (MathWorks, USA), and p-values < 0.05 were considered to be statistically significant.

## Results

CT-aBMD was successfully measured in 976/978 hips (99.8%). In one of the failed cases, CT-aBMD was not measured because of an error in femoral segmentation; in the other case, CT-aBMD was not measured because of an error in landmark selection.

### Comparison between DXA-BMD and CT-aBMD

**Fig. 2:**
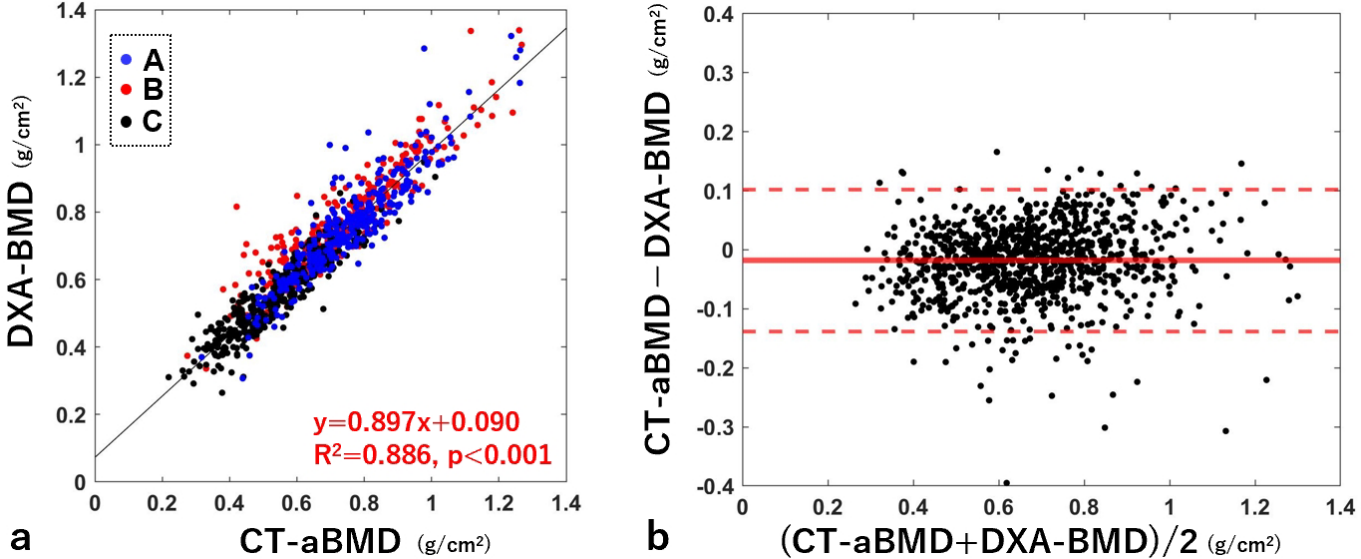
a) Correlation plots between CT-aBMD and DXA-BMD and b) the corresponding Bland–Altman plot. For a), the blue, red, and black dots indicate cases from institutions A, B, and C, respectively. The black line indicates the regression line, and the red text indicates the regression equation, coefficient of determination, and p-value. The thick red line in b) indicates the mean value of the plots, and the thin red dotted lines indicate the 95% limits of agreement. aBMD, areal bone mineral density; DXA, dual-energy X-ray absorptiometry.

A significant correlation was observed between CT-aBMD and DXA-BMD. The correlation coefficient was 0.941 (p < 0.001) (Figure 2a). In the Bland–Altman analysis, the mean difference between CT-aBMD and DXA-BMD was -0.02 g/cm^2^ (Figure 2b). The median absolute error was 0.04 g/cm^2^ (IQR 0.02 to 0.06).

Across institutions, the absolute error was significantly larger for institution B (0.05 g/cm^2^) than for institutions A (0.03 g/cm^2^) and C (0.03 g/cm^2^) (p = 0.002 and p < 0.001, respectively (Kruskal–Wallis test); Supplementary Figure aa). Across CT manufacturers, the absolute error for GE scanner (0.03 g/cm^2^) was significantly smaller than those for Toshiba (0.04 g/cm^2^) and Siemens (0.05 g/cm^2^) scanners (p = 0.004 and p = 0.003, respectively (Kruskal–Wallis test); Supplementary Figure ab).

### ROC curve analysis

**Fig. 3:**
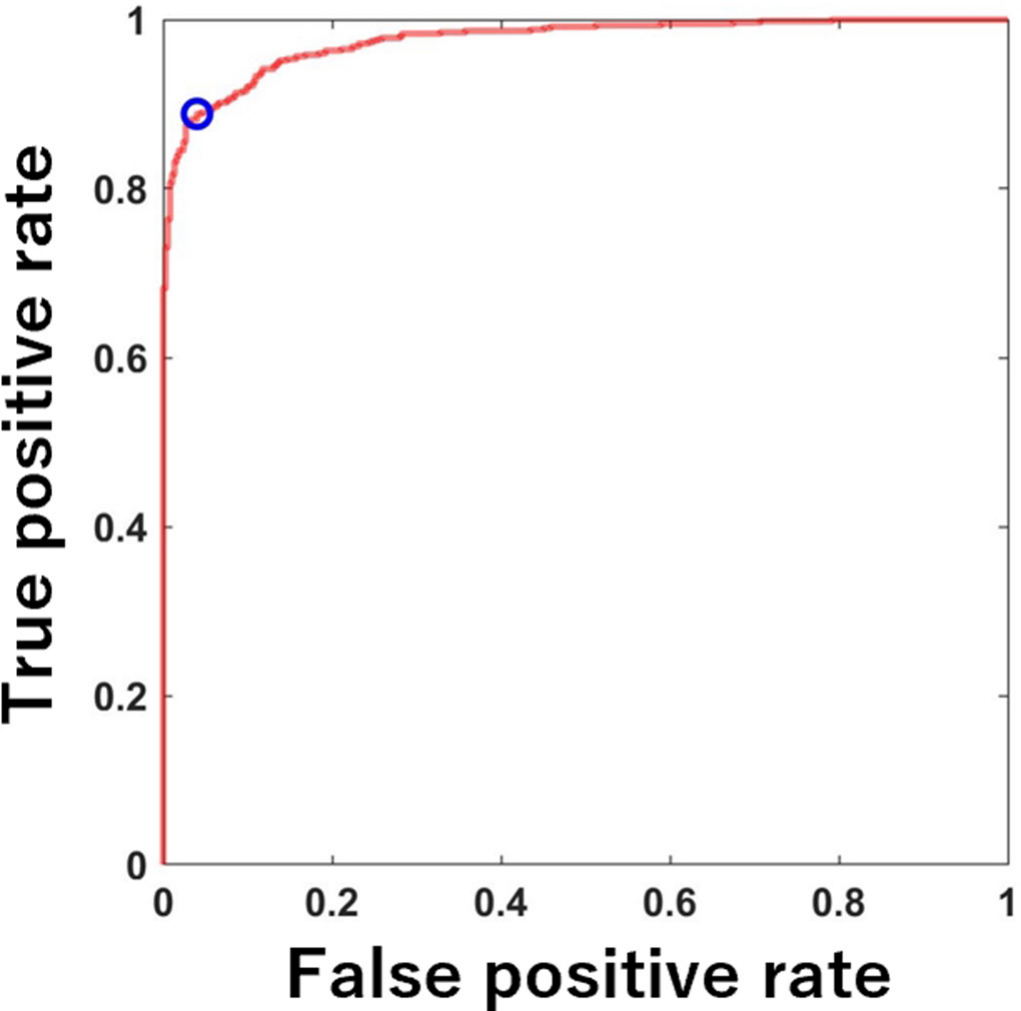
Receiver operating characteristic curve analysis for diagnosing osteoporosis using CT-aBMD. The blue circle indicates the optimal cutoff point. aBMD, areal bone mineral density.

In the ROC analysis, the AUC to diagnose osteoporosis was 0.976 (Figure 3). The diagnostic sensitivity and specificity were 88.9% and 96%, respectively, with a cutoff value of 0.625 g/cm^2^.

### Validation of the landmark detection model

**Fig. 4:**
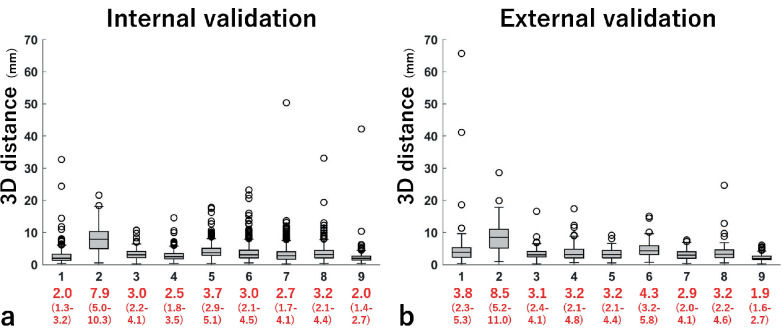
Results of the 3D distance between the landmarks selected automatically and manually for a) internal and b) external validations. Red numbers indicate the median in millimetres (interquartile range) for landmarks 1 to 9 (landmark numbers refer to Figure 1c).

The median 3D distance ranged from 2.0 mm to 7.9 mm for internal validation (Figure 4a). By contrast, the 3D distance ranged from 1.9 mm to 8.5 mm for external validation (Figure 4b). In the measurements of the hip angles from the landmarks, the median absolute angular difference calculated from manually and automatically selected landmarks was less than 2° for abduction and flexion for both internal and external validations. Moreover, the errors were 2.2° and 2.5° for the internal and external validations, respectively (Supplementary Figures ba and bb). In the Bland–Altman analysis for CT-aBMD measurements using manually or automatically selected landmarks, the mean differences in CT-aBMD were -0.002 g/cm^2^ and -0.007 g/cm^2^ for the internal (Supplementary Figure bc) and external (Supplementary Figure bd) validations, respectively. The median absolute errors were 0.005 g/cm^2^ (IQR 0.002 to 0.008) and 0.007 g/cm^2^ (IQR 0.004-0.013) for the internal and external validations, respectively.

## Discussion

In this study, a pipeline consisting of three deep-learning models was applied to develop an open-source system that automatically measures the BMD of the proximal femur from CT images.^26^ A very strong correlation was observed between CT-aBMD and DXA-BMD (r = 0.941). In the ROC analysis for diagnosing osteoporosis, the AUC was 0.976. Collectively, our results indicate that accurate DXA-BMD measurement and diagnosis of osteoporosis can be performed from clinical CT images using the system developed in this study.

### Hips with measurement error

In the majority of hips (976 hips, 99.8%), CT-aBMD was automatically measured using the developed system. However, the system failed in quantifying the CT-aBMD in two cases. One case had an extremely low BMD (0.274 g/cm^2^, calculated as a T-score of −5.0)^1^ that likely led to errors in femoral segmentation. In addition, when the landmarks were manually selected for these two cases as a post-hoc analysis, the hip angles relative to the CT table were 20.2° and 34.3° for flexion, 32.4° and 44.8° for adduction, and 61.7° and 73.8° for internal rotation. During CT, patient positioning was excessive and beyond the range of data augmentation performed in the training dataset for segmenting the femur and detecting the landmarks; thus, errors likely occurred in these two cases.

### Accuracy of the landmark detection model

The 3D distance errors in some landmarks were relatively large (e.g. 7.9 mm to 8.5 mm for 5 cm distal from the lesser trochanter; Figures 4a and 4b). Conversely, errors in measuring the CT-aBMD were small (< 0.01 g/cm^2^; Figures 4c and 4d). Previous studies have reported the importance of defining the distal border of the ROI in the BMD measurement, because a 1 mm change results in a BMD change of 0.54% to 0.68%.^27,28^ Further, the errors in angle measurements to account for the variance in patient positioning during CT were small (Figures 4c and 4d), especially for abduction and flexion, which independently lead to errors in BMD measurements.^29^ As the distal border of the ROI was defined based on the tip of the lesser trochanter that had small errors in the landmark detection model (1.9 mm to 2.0 mm) and as hip abduction and flexion were accurately compensated, the CT-aBMD was likely quantified with less error.

### Comparisons with previous reports

Previous studies have used commercially available software to measure the BMD of the proximal femur from CT images and reported very strong correlations with DXA-BMD, with correlation coefficients of 0.91 to 0.95.^11-15^ In the ROC curve analysis, commercially available software can diagnose osteoporosis with sensitivity and specificity ranging from 83% to 99%.^12,14,15^

In this study, the correlation coefficient between CT-aBMD and DXA-BMD was 0.941, and the sensitivity and specificity for diagnosing osteoporosis were 88.9% and 96%, respectively. Interestingly, the cutoff value to diagnose osteoporosis was 0.625 g/cm^2^, strikingly similar to the value set for female Japanese patients using Hologic’s DXA,^1^ supporting the validity of our system in representing the DXA-BMD. Compared with the previous system requiring manual landmark selection, the correlation coefficient between CT-aBMD and DXA-BMD in the present study (r = 0.941) was comparable to that of another previous study analyzed for 75 cases (r = 0.950).^17^ Thus, the automated system developed herein had accuracy comparable to that of the previous method requiring manual input.

Overall, although areal BMD measured by our system and areal BMD measured by the commercially available software were not directly compared in this study, our results based on a large sample size from a multicentre study support the finding that the developed software has similar accuracy to the commercially available software in predicting DXA-BMD and screening for osteoporosis.

### Comparison across institutions and CT manufacturers

Statistically significant differences were found in the absolute differences between CT-aBMD and DXA-BMD across institutions and CT manufacturers. Further investigation is necessary to clarify the causality behind the differences because direct comparison is not possible due to the variations in patient backgrounds across institutions and CT manufacturers. However, errors in predicting DXA-BMD using CT-aBMD were still small for the institution and manufacturer that had larger errors (0.05 g/cm^2^) and had an excellent ability to detect osteoporosis.

### Application of the developed system to clinical evaluation

The developed open-source system can be used to evaluate the proximal femur BMD and opportunistically screen for osteoporosis of patients who have acquired hip CT images for surgical planning of hip arthroplasty (e.g. CT-based navigation system and CT-based robotic surgery). The occurrence of periprosthetic fracture is an issue after cementless hip arthroplasty, so our system can help surgeons to select the stem implant type, such as cementless or cemented.^30^

As a calibration phantom is not always included in CT images acquired for clinical investigation, additional experiment without phantom calibration (i.e. phantom-less) was performed as a post-hoc analysis. In the analysis, the correlation coefficient between CT-aBMD and DXA-BMD was 0.927 (Supplementary Figure ca), and the AUC for diagnosing osteoporosis was 0.967 (Supplementary Figure cb). The diagnostic sensitivity and specificity were 90.2% and 90.7%, respectively, with the cutoff value of 0.624 g/cm^2^. As the AUC, sensitivity, and specificity remained high, we believe that phantoms are not always necessary, and asynchronous calibration may be useful to screen CT images with notable differences in HUs.^13^ Further, we plan to apply the system to abdominal CT images acquired without a calibration phantom, and clarify the possibility of using the system for diagnosing osteoporosis from such images in a future study.

This study had some limitations. First, while our system was verified on a multicentre study using a large sample size (978 hips) in which the patient demographics, CT imaging, and reconstruction settings varied, errors may occur if CT images were acquired under a different protocol and/or under different CT models. Applying our system and confirming its ability to accurately measure the BMD and screen for osteoporosis for CT images acquired under such conditions would be interesting. Second, patients with metal implants were excluded from the analysis because these artifacts may affect the results. As our next step, we intend to accurately measure the BMD of such patients and use a deep-learning method to reduce metal artifacts^31^ to provide adequate treatment of osteoporosis and prevent loosening of implants.

In conclusion, a fully automated system to predict the DXA-BMD from CT images was developed and verified in a multicentre study of 978 hips. A very strong correlation was found between CT-aBMD and DXA-BMD, with a correlation coefficient of 0.941. The median absolute error in estimating the DXA-BMD was 0.04 g/cm^2^. In the ROC analysis to diagnose osteoporosis, the AUC showed a high performance of 0.976. As the system can accurately measure BMD from CT images using open-source models,^26^ clinicians can opportunistically screen osteoporosis from hip CT images and select the type of implant for hip surgery using the developed system.
